# The Long and the Short of PTEN in the Regulation of Mitophagy

**DOI:** 10.3389/fcell.2020.00299

**Published:** 2020-05-13

**Authors:** Liming Wang, Guang Lu, Han-Ming Shen

**Affiliations:** ^1^Department of Physiology, Yong Loo Lin School of Medicine, National University of Singapore, Singapore, Singapore; ^2^Faculty of Health Sciences, University of Macau, Macau, China

**Keywords:** mitophagy, PINK1, Parkin, BNIP3, PTEN, PTEN-L

## Abstract

Mitophagy is a key mitochondrial quality control mechanism for effective and selective elimination of damaged mitochondria through the autophagy-lysosome machinery. Defective mitophagy is associated with pathogenesis of important human diseases including neurodegenerative diseases, heart failure, innate immunity, and cancer. In the past two decades, the mechanistic studies of mitophagy have made many breakthroughs with the discoveries of phosphatase and tensin homolog (PTEN)-induced kinase protein 1 (PINK1)-parkin-mediated ubiquitin (Ub)-driven pathway and BCL2/adenovirus E1B 19 kDa protein-interacting proteins 3 (BNIP3)/NIX or FUN14 domain containing 1 (FUNDC1) mitochondrial receptor-mediated pathways. Recently, several isoforms of dual phosphatase PTEN, such as PTEN-long (PTEN-L), have been identified, and some of them are implicated in the mitophagy process via their protein phosphatase activity. In this review, we aim to discuss the regulatory roles of PTEN isoforms in mitophagy. These discoveries may provide new opportunities for development of novel therapeutic strategies for mitophagy-related diseases such as neurodegenerative disorders via targeting PTEN isoforms and mitophagy.

## Introduction

Autophagy is an evolutionarily conserved process to degrade or recycle intracellular materials through lysosomes or vacuoles (Mizushima, 2018). In mammalian cells, there exist three different types of autophagy: macroautophagy, microautophagy, and chaperone-mediated autophagy (CMA). Among them, macroautophagy (referred to as autophagy hereafter) is the most well-studied form, which is orchestrated by a group of proteins encoded by autophagy-related-genes (*ATGs*) and characterized by the formation of double-membraned autophagosomes (Zachari and Ganley, 2017; Dikic and Elazar, 2018; Mizushima, 2018). The formation of autophagosomes can be briefly divided into three main steps: (1) The initiation step is regulated by unc51-like activating kinase 1 (ULK1) complex comprised of ULK1, ATG13, FIP200, and ATG101 to form the phagophore; (2) the vesicle nucleation step is regulated by Beclin1-ATG14 and Vps34/class III phosphatidylinositol 3-kinases (PI3K) complex to generate phosphatidylinositol 3-phosphate (PI3P); and (3) the vesicle elongation step is mediated by two ubiquitination conjugation systems, ATG12-ATG5-ATG16L1 and LC3-PE (phosphatidylethanolamine) systems, as well as ATG9-containing vesicles to form the autophagosomes (Mizushima et al., 2011; Hurley and Young, 2017; Lahiri et al., 2019). Autophagy can be either a general non-selective process to randomly uptake cargos for degradation (bulk autophagy) or a selective process to remove or degrade specific organelles, aggregated proteins, DNA, and/or invading pathogens (selective autophagy). Up to date, several types of selective autophagy have been recognized, including mitophagy, ribophagy, xenophagy, reticulophagy, lysophagy, and aggrephagy (Rogov et al., 2014; Kirkin, 2020).

Among them, mitophagy represents the most well-studied form of selective autophagy to degrade dysfunctional or superfluous mitochondria through the autophagy-lysosome machinery, which is regulated by multiple factors with distinct posttranslational modifications (Montava-Garriga and Ganley, 2020; Wang et al., 2020). The phenomenon of mitophagy was first described by Christian De Duve and Robert Wattiaux in 1966 when they observed that mitochondria were engulfed by autophagic vacuoles (De Duve and Wattiaux, 1966). The term of “mitophagy” was coined by John J. Lemasters to distinguish this selective autophagy that degrades mitochondria from the bulk autophagy (Lemasters, 2005). Mitophagy is usually initiated by an “eat me” signal, such as labeling damaged mitochondria with ubiquitin (Ub) or autophagy receptors (Harper et al., 2018; Pickles et al., 2018; Wang et al., 2020). Owing to its critical role in maintaining mitochondrial homeostasis and close implication in multiple human diseases, such as Parkinson’s disease (PD) and Alzheimer’s disease (AD) (Williams and Ding, 2018; Lou et al., 2019), the machinery of mitophagy has drawn substantial attention in the past two decades. The discoveries of PINK1-Parkin-mediated Ub-driven pathway and BNIP3/NIX or FUNDC1 receptor-mediated pathways represent the milestones in the mitophagy field. In this review, we will discuss some of these key factors, especially the newly identified protein phosphatase, in the regulation of mitophagy.

## PINK1-Parkin-Mediated Ubiquitin-Driven Mitophagy

One breakthrough in the understanding of the molecular mechanisms of mitophagy is the discovery of PINK1-Parkin-mediated pathway (Narendra et al., 2008, 2010; Vives-Bauza et al., 2010). PINK1 (encoded by the *PARK6* gene) is a serine/threonine kinase, which was identified in 2001 (Unoki and Nakamura, 2001) and contains a mitochondrial targeting sequence (MTS) at its N-terminus as well as an outer mitochondrial localization signal (OMS) next to the transmembrane domain (TMD) (Okatsu et al., 2015a). Two homozygous mutations, including G→A in transition in exon 4 and G→A transitions in exon 7, in *PINK1* were found in autosomal recessive early onset familial forms of PD patients (Valente et al., 2004). Parkin (encoded by the *PARK2* gene) is an E3 Ub ligase, which was identified in 1998 and was named “Parkin” due to its important roles in the pathogenesis of autosomal recessive juvenile parkinsonism (AR-JP) (Kitada et al., 1998; Lucking et al., 1998; Abbas et al., 1999). Parkin contains a Ub-like (UBL) domain, a classic RING (RING1) domain, three zinc-coordinating domains termed in between RING (IBR) domain, a RING2 domain, and a RING0 domain that is a Parkin unique domain (Hristova et al., 2009; Trempe et al., 2013; Walden and Muqit, 2017). Numerous studies have reported that PINK1 and Parkin work in the same pathway to remove dysfunctional mitochondria and to maintain mitochondrial homeostasis, with the well-established feedforward model of PINK1-Parkin mitophagy activation (Harper et al., 2018; Pickles et al., 2018; Wang et al., 2020).

When mitochondria are healthy, PINK1 is constantly maintained at a low level due to mitochondrial import, protease cleavage, and proteasome degradation (Jin et al., 2010; Deas et al., 2011; Lazarou et al., 2012; Sekine et al., 2019). Upon mitochondrial damage and depolarization, PINK1 is rapidly accumulated on the outer mitochondrial membrane (OMM) and activated through dimerization and autophosphorylation (Okatsu et al., 2012, 2013; Aerts et al., 2015; Rasool et al., 2018). Therefore, PINK1 acts as a mitochondrial damage sensor to initiate mitophagy. Once activated, PINK1 phosphorylates mitochondrial pre-existing Ub at Ser 65 (pSer65-Ub) (Kane et al., 2014; Kazlauskaite et al., 2014; Koyano et al., 2014; Shiba-Fukushima et al., 2014). pSer65-Ub serves as a key receptor to recruit Parkin from cytosol to mitochondria through direct binding (Shiba-Fukushima et al., 2014; Okatsu et al., 2015b). Binding to pSer65-Ub releases the UBL domain of Parkin from its RING1 domain (Sauve et al., 2015; Wauer et al., 2015a; Aguirre et al., 2017), which promotes the phosphorylation of the UBL domain by PINK1 at Ser 65 (pSer65-Parkin) (Kondapalli et al., 2012; Shiba-Fukushima et al., 2012; Wauer et al., 2015a; McWilliams et al., 2018). Subsequently, the phospho-UBL domain rebinds to the RING0 domain of Parkin to release the catalytic RING2 domain to achieve full activation (Gladkova et al., 2018; Sauve et al., 2018). Activated Parkin then conjugates more Ub onto OMM proteins for PINK1 phosphorylation, which mediates further rounds of Parkin translocation to mitochondria; thus, PINK1, pSer65-Ub, and Parkin form a positive feedforward amplification loop to initiate mitophagy.

Another important function of pSer65-Ub is to recruit autophagy receptors, such as NDP52 (CALCOCO2) and Optineurin (OPTN) to damaged mitochondria, a process that is TANK-binding kinase 1 (TBK1) dependent (Heo et al., 2015; Lazarou et al., 2015; Richter et al., 2016). TBK1 is a serine/threonine kinase and phosphorylates these autophagy receptors to promote their binding ability to various Ub chains (Heo et al., 2015; Richter et al., 2016). Interestingly, activation of TBK1 also requires OPTN binding to Ub chains in the presence of PINK1 and Parkin (Heo et al., 2015; Richter et al., 2016). In the prevailing model of mitophagy, after binding to the pSer65-Ub chains, OPTN and/or NDP52 recruit phagophore onto mitochondria by directly binding to LC3 through their LC3-interacting regions (LIR motifs) (Gatica et al., 2018; Palikaras et al., 2018). However, emerging studies suggest that LC3/GABARAP family proteins are dispensable in the selective recognition of damaged mitochondria, based on the observation that, in LC3/GABARAP knockout cells, mitochondria can still be engulfed by autophagosomes (Itakura et al., 2012; Nguyen et al., 2016; Padman et al., 2019). One very recent study has highlighted the role of NDP52 to recruit ULK1 complex to damaged mitochondria (Vargas et al., 2019). NDP52 directly interacts with FIP200 in a TBK1-dependent manner to recruit ULK1 complex, leading to autophagosome biogenesis on damaged mitochondria and initiation of autophagy machinery.

Interestingly, besides PINK1-mediated pSer65-Ub, several other PINK1-independent phosphorylation sites of Ub have been identified, including pThr7-Ub, pSer20-Ub, and pSer57-Ub (Wauer et al., 2015b). Among them, pSer57-Ub has been reported to hyperactivate Parkin (George et al., 2017). Obviously, more studies are needed to understand the functional implication of such Ub phosphorylation in mitophagy. In addition to Ub and Parkin as described above, a number of additional PINK1 substrates have been reported. For instance, PINK1 phosphorylates mitofusin 2 (MFN2) at Thr 111 and Ser 442, leading to Parkin mitochondrial recruitment through promoting the interaction between MFN2 and Parkin, suggesting that MFN2 may serve as a mitochondrial receptor for Parkin (Chen and Dorn, 2013). However, another study indicates that MFN2 antagonizes mitophagy through tethering mitochondria and endoplasmic reticulum (ER) and limiting the accessibility of other mitochondrial proteins to PINK1 and Parkin (McLelland et al., 2018). It is known that some OMM proteins such as MFN2 undergo ubiquitination and proteasomal degradation at the beginning of the mitophagy (Tanaka et al., 2010; Ding et al., 2012; McLelland et al., 2018). Therefore, it is possible that such a process may facilitate mitophagy by removing the barrier among PINK1, Parkin, and other mitochondrial proteins. PINK1 can also phosphorylate Miro (also called RhoT) at Ser156, which recruits Parkin onto mitochondria and results in ubiquitination and proteasomal degradation of Miro, and thus blocking mitochondrial motility (Wang et al., 2011; Shlevkov et al., 2016). Interestingly, a recent report found that Miro, through direct protein–protein interaction, recruits Parkin at healthy mitochondria independent of PINK1, and such pre-existing Parkin is essential for Parkin further recruitment and activation upon mitochondrial damage in a PINK1-dependent manner (Safiulina et al., 2019). In addition, in a phosphoproteomic screening study for PINK1 substrates, Lai and colleagues reported that the phosphorylation of Rab GTPases such as Rab8A at the conserved Ser 111 is indirectly regulated by PINK1, and this phosphorylation can block the phosphorylation of Rab8A at Thr72 by leucine-rich repeat kinase 2 (LRRK2), suggesting the interplay of PINK1 with other PD-related genes (Lai et al., 2015; Vieweg et al., 2019). Thus, identification of more PINK1 substrates will not only provide new insights into the molecular mechanisms of PINK1-Parkin-mediated mitophagy but also provide deeper understanding of the molecular mechanisms of important neurodegenerative disorders such as PD.

## BNIP3/NIX (BNIP3L)-Mediated Mitophagy

BNIP3, a member of prodeath BCL2 family proteins, was first found as an E1B 19-kDa interacting proteins (Boyd et al., 1994). NIX (also named BNIP3L) is a homolog of BNIP3 with ∼55% identical similar amino acid sequence (Matsushima et al., 1998). Both proteins contain an atypical BCL2-homology 3 (BH3) domain and C-terminal TMD, which is essential for their proapoptotic activity and mitochondrial localization (Yasuda et al., 1998; Imazu et al., 1999). Moreover, BNIP3 and NIX both contain an identical LIR motif, which makes them to interact with LC3s/GABARAP subfamilies and recruit autophagosomes to sequester damaged mitochondria, especially under hypoxia conditions (Novak et al., 2010; Hanna et al., 2012; Birgisdottir et al., 2013). Under hypoxia, the expression of BNIP3 and NIX are increased through the transcriptional regulation of hypoxia-inducible factor 1α (HIF-1α) or FOXO3 (Sowter et al., 2001; Mammucari et al., 2007; Zhang et al., 2008). Mutation of the LIR motif abolishes the interaction of BNIP3/NIX with LC3 and thereby attenuates mitochondrial clearance (Novak et al., 2010; Hanna et al., 2012; Zhu et al., 2013), while phosphorylation of the LIR motif enhances the interaction with LC3 and promotes mitophagy (Zhu et al., 2013; Rogov et al., 2017). However, the kinase(s) and phosphatase(s) regulating this phosphorylation of LIR remain to be identified.

It should be noted that NIX, but not BNIP3, plays an important role in the development of reticulocytes through the regulation of mitophagy. Mitochondria were not cleared in reticulocytes when NIX is deficient (Diwan et al., 2007; Schweers et al., 2007; Zhang and Ney, 2008; Zhang J. et al., 2012). Interestingly, treatment with mitochondrial uncoupling agents could restore the removal of mitochondria in the absence of NIX, suggesting that the regulatory effect of NIX on mitophagy was probably due to its role in regulating mitochondrial depolarization (Sandoval et al., 2008; Zhang and Ney, 2008). However, there is still no direct evidence to show that NIX could cause mitochondrial depolarization, and further studies are thus needed.

Intriguingly, several studies have revealed the crosstalk between BNIP3/NIX receptor-mediated pathway and PINK1-Parkin-mediated pathway. For instance, both BNIP3 and NIX can promote Parkin mitochondrial recruitment (Ding et al., 2010; Lee et al., 2011), while NIX can also be ubiquitinated by Parkin to promote autophagy receptor recruitment to damaged mitochondria (Gao et al., 2015). In addition, BNIP3 is able to inhibit PINK1 proteolytic degradation and stabilize PINK1 on OMM to facilitate Parkin mitochondrial recruitment and mitophagy (Zhang et al., 2016). These findings suggest that these pathways cooperate with each other to ensure efficient mitophagy.

## FUNDC1-Mediated Mitophagy

FUNDC1 is another important hypoxia-induced mitophagy receptor (Liu et al., 2012). As a mitochondrial outer membrane protein, FUNDC1 contains three TMDs and an LIR motif in its N-terminus exposed to the cytosol that interacts with LC3 to recruit autophagosome (Liu et al., 2012; Wu et al., 2016). Mutation or deletion of LIR motif of FUNDC1 significantly reduces or blocks mitophagy (Liu et al., 2012). Similar to the cases of other mitophagy key factors, the activity of FUNDC1 is also regulated by phosphorylation and dephosphorylation. Under normal conditions, FUNDC1 is phosphorylated by Src and CK2 at the sites of Tyr18 and Ser13, which blocks the interaction of FUNDC1 with LC3 (Liu et al., 2012; Chen et al., 2014). Another study showed that FUNDC1 can be phosphorylated by ULK1 at Ser17 to promote mitophagy (Wu et al., 2014). However, upon induction of hypoxia, Src and CK2 are inhibited, then phosphoglycerate mutase family member 5 (PGAM5), one unique mitochondrial phosphatase, dephosphorylates FUNDC1 at Ser13, which in turn promotes the interaction between FUNDC1 and LC3 to facilitate mitophagy (Chen et al., 2014). Interestingly, the same group reported that FUNDC1 is accumulated at the ER-mitochondrial contact site in response to hypoxia, which is essential for the mitochondrial recruitment of DRP1 to facilitate mitochondrial fission prior to mitophagy (Wu et al., 2016).

## Canonical PTEN (PTEN-Short) as a Negative Regulator of Mitophagy

**FIGURE 1:**
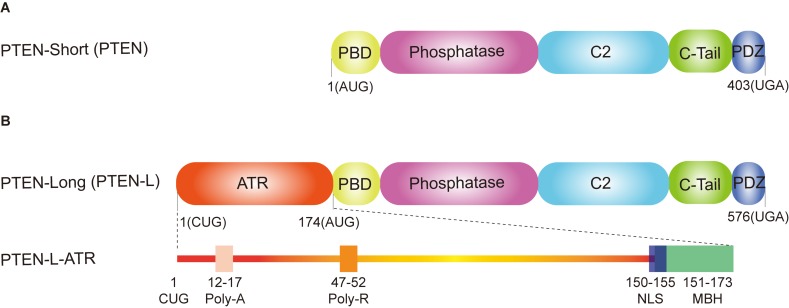
Domain structure of phosphatase and tensin homolog (PTEN) isoforms. **(A)** PTEN-short (canonical PTEN), translated from an AUG start codon, contains five functional domains: a N-terminal PtdIns (4,5) P2 (PIP2)-binding domain (PBD), a dual phosphatase domain, a C2 domain, a C-tail domain, and PDZ-binding motif. **(B)** PTEN-long (PTEN-L) is translated from a CUG start codon upstream from the classic AUG start codon. In addition to the same five functional domains with the canonical PTEN, PTEN-L contains an alternatively translated region (ATR) adding 173 amino acids at the N-terminus. The extended ATR is composed of a secreted polyalanine signal sequence (Poly-A, residues 12–17), a cell permeable polyarginine motif (Poly-R, residues 47–52), a nuclear localization sequence (NLS, QKKPRH, residues 150–155) as well as a membrane-binding α-helix (MBH, residues 151–173).

PTEN is a powerful tumor suppressor with both lipid phosphatase and protein phosphatase activity, which was identified in 1997 (Li and Sun, 1997; Li et al., 1997; Steck et al., 1997). PTEN contains 403 amino acids with a N-terminal phosphatidylinositol (4,5)-bisphosphate [PI(4,5)P2]-binding domain (PBD), a catalytic phosphatase domain, a C2 domain, a C-tail domain, and a PDZ-binding motif (Figure 1A; Lee et al., 1999). Loss of PTEN leads to cancer, neurological disorders, metabolic diseases, and tissue homeostasis defects (Backman et al., 2001; Kwon et al., 2006; Chen et al., 2018; Lee et al., 2018). PTEN is also vital for embryonic development, as its homozygous deletion causes lethality in mice (Di Cristofano et al., 1998; Stumpf and den Hertog, 2016). All these findings reveal that PTEN’s function is not only important for tumor suppression but also vital for other biological processes.

The probably most important function of PTEN is to block the activation of pro-oncogenic class I PI3K–AKT–mTOR signaling pathway through its lipid phosphatase activity (Cantley and Neel, 1999). PI3K phosphorylates PI(4,5)P2 to generate phosphatidylinositol (3,4,5)-trisphosphate [PI(3,4,5)P3], which recruits AKT at the cell membrane, and then AKT is phosphorylated via PDK1 and mTORC2 to indirectly activate mTORC1 (King et al., 2015). PTEN opposes this pathway through dephosphorylating PI(3,4,5)P3 to PI(4,5)P2 via its lipid phosphatase activity, leading to reduced AKT phosphorylation and inactivation (Worby and Dixon, 2014). Thus, the phosphorylation level of AKT has been widely used as an indicator for PTEN activity.

Due to the inhibitory effects of PTEN on the PI3K–AKT–mTOR signaling pathway, several studies have shown that PTEN can positively regulate autophagy (Arico et al., 2001; Ueno et al., 2008; Cai et al., 2018). Intriguingly, two independent groups reported that inhibition of AKT signaling impaired PINK1 accumulation, Parkin recruitment, and subsequent efficient mitophagy in response to mitochondrial depolarization (McCoy et al., 2014; Soutar et al., 2018). However, the role of PTEN in the regulation of mitophagy is still largely unclear. Harper and colleagues reported that RAB7A could be directly phosphorylated by TBK1 at Ser 72 (pSer72-RAB7A) to facilitate the efficient recruitment of ATG9A vesicles to damaged mitochondria and promote PINK1-Parkin-mediated mitophagy, and non-phosphorylated RAB7A failed to support this process (Heo et al., 2018). Importantly, PTEN has been found to dephosphorylate pSer72-RAB7A via its protein phosphatase activity (Shinde and Maddika, 2016; Hanafusa et al., 2019), thus suggesting a potential role of PTEN in regulating mitophagy. A more direct study showed that deletion of PTEN increased MFN2 expression and rescued mitophagic flux via the AMP-activated protein kinase (AMPK)–cAMP response element-binding protein (CREB) pathways (Li et al., 2019). Interestingly, both PTEN and MFN2 have a distribution at ER-mitochondrial contact site (de Brito and Scorrano, 2008; Bononi et al., 2013; Naon et al., 2016). As discussed above, MFN2 can be phosphorylated by PINK1 and serves as a mitochondrial receptor for Parkin (Chen and Dorn, 2013). Moreover, phosphorylated MFN2 dissociates mitochondria from ER to initiate mitophagy (McLelland et al., 2018). Thus, it will be interesting to explore whether PTEN can dephosphorylate MFN2 at the ER-mitochondrial contact site to suppress mitophagy. In addition, overexpression of PTEN inhibits mitophagy via blockage of Toll-like receptor 4 (TLR4)–c-JUN N-terminal kinase (JNK)–BNIP3 pathway (Li M. et al., 2018).

Moreover, several *in vivo* studies have highlighted that PTEN deletion in dopamine neurons provides neuroprotective effects in both genetic and neurotoxin-induced PD mouse models (Diaz-Ruiz et al., 2009; Domanskyi et al., 2011; Zhang Y. et al., 2012). Another study showed that the protein level of PTEN is significantly increased in neurotoxin 1-methyl-4-phenyl-1,2,3,6-tetrahydropyridine-hydrochloride (MPTP)-treated mice and 1-methyl-4-phenylpyridinium (MPP^+^)-treated SH-SY5Y cells, leading to enhanced neurotoxicity and apoptosis (Zhao et al., 2020). In addition, inhibition of PTEN is able to attenuate amyloid-β (Aβ)-induced synaptic toxicity and rescue cognitive function in AD models (Knafo et al., 2016). Consistently, a PTEN inhibitor, bisperoxovanadium-pic [bpV(pic)], provides neuroprotective effects in Aβ-induced neurotoxicity in a human neuroblastoma cell model (Liu et al., 2017). Apparently, more studies are needed to explore whether the above processes are due to the regulative effects of PTEN on mitophagy.

## Novel PTEN-L (PTEN-Long) as a Brake of Mitophagy

PTEN-L is the first characterized isoform of canonical PTEN, which was identified in 2013 (Hopkins et al., 2013). PTEN-L and PTEN shares the same mRNA, but PTEN-L translates from a non-AUG start codon (CUG start codon), adding an alternatively translated region (ATR) at the N-terminus of PTEN (Hopkins et al., 2013). PTEN-L can be secreted from one cell and taken up by other neighboring cells to inhibit PI3K–AKT signaling pathway both *in vitro* and *in vivo* (Hopkins et al., 2013). Intriguingly, Liang et al. reported that PTEN-L (also termed as PTENα) is a mitochondrial protein to regulate mitochondrial energy metabolism (Liang et al., 2014). They found that somatic deletion of PTEN-L resulted in much smaller mitochondria with irregular shape and led to mitochondrial depolarization (Liang et al., 2014). It is known that, in addition to the same domains with canonical PTEN (PTEN-short), the extended ATR of PTEN-L contains a secreted polyalanine signal sequence (Poly-A), a cell permeable polyarginine motif (Poly-R), a nuclear localization sequence (NLS, QKKPRH) as well as a membrane-binding α-helix (MBH) (Figure 1B; Hopkins et al., 2013; Malaney et al., 2013; Masson et al., 2016; Shen et al., 2019). In addition, most parts of the ATR are intrinsically disordered and probably contain various postmodification sites and protein-binding motifs (Malaney et al., 2013; Masson et al., 2016), indicating that PTEN-L may modify distinct substrates compared with PTEN.

Recently, our group has revealed that PTEN-L functions as a protein phosphatase for Ub and antagonizes the PINK1-Parkin-mediated mitophagy pathway (Wang et al., 2018a, b). First, topology assay and immunogold electron microscopy revealed that a significant proportion of PTEN-L was associated with the mitochondrial outer membrane. Second, PTEN-L overexpression blocked mitophagy induced by mitochondrial damage agents including carbonyl cyanide 3-chlorophenylhydrazone (CCCP), combination of oligomycin and antimycin A (O/A), and valinomycin, whereas PTEN-L knockout accelerated mitophagic flux. Third, PTEN-L overexpression was able to strongly prevent Parkin mitochondrial recruitment, autoubiquitination, and subsequent activation of its E3 ligase activity. Finally, PTEN-L could dephosphorylate various types of pSer65-Ub chains *in vivo* and *in vitro* via its protein phosphatase activity but independent of its lipid phosphatase activity, leading to the disruption of the feedforward amplification loops formed by PINK1, Parkin, and pSer65-Ub chains. Since Ub modification is a vital posttranslational process in mitophagy, deubiquitinating enzymes (DUBs) become potential regulators to maintain the mitochondrial homeostasis, especially in the PINK1-Parkin-mediated Ub-driven mitophagy pathway. There are more than 100 putative DUB genes in humans, which can be grouped into two classes: cysteine proteases and metalloproteases. Among them, ubiquitin-specific proteases (USPs), which are encoded by 58 different genes, such as USP30, USP15, and USP8, have been widely studied in the field of mitophagy (Bingol et al., 2014; Cornelissen et al., 2014; Durcan et al., 2014; Marcassa et al., 2018; Ordureau et al., 2020). Recently, USP36 has been reported as a positive regulator of mitophagy; knockdown of USP36 impairs Parkin mitochondrial translocation, leading to blockage of mitophagy (Geisler et al., 2019). Interestingly, they also found that the protein level of PTEN-L was increased after USP36 knockdown, which was associated with reduced pSer65-Ub level and consistent with our findings (Geisler et al., 2019).

Intriguingly, Li et al. demonstrated that PTEN-L promotes mitophagy through interaction with Parkin by its MBH motif to promote Parkin self-association and mitochondrial localization (Li G. et al., 2018). Further studies are thus needed to examine the precise role of PTEN-L in this pathway and more importantly to explore whether PTEN-L is implicated in the pathology of mitophagy-related diseases, such as PD and AD.

## Conclusion and Future Directions

**FIGURE 2:**
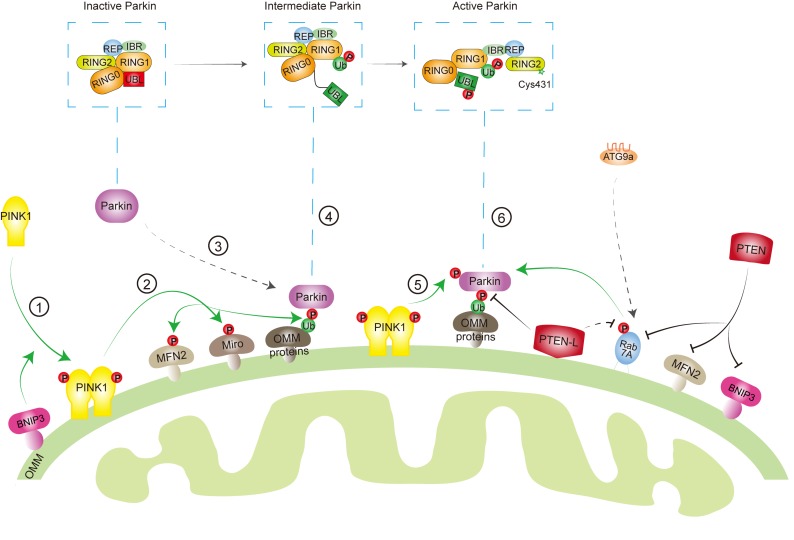
Key effectors involved in mitophagy machinery. When mitochondria are healthy, phosphatase and tensin homolog-induced kinase protein 1 (PINK1) is imported into the mitochondria, cleaved by protease, and degraded by proteasome, while Parkin keeps in an inactive conformation in the cytosol through intradomain–domain interactions. Upon mitochondrial damage or depolarization, PINK1 is stabilized and activated at the outer mitochondrial membrane (OMM) ➀, which leads to the phosphorylation of its downstream targets, such as ubiquitin (Ub) ➁. Parkin has a high affinity to phosphorylated Ub (pSer65-Ub), which recruits Parkin from cytosol to mitochondria ➂. Several other factors, such as mitofusin 2 (MFN2), Miro, Rab7A, as well as BCL2/adenovirus E1B 19 kDa protein-interacting proteins 3 (BNIP3) are also involved in Parkin mitochondrial recruitment. Binding to pSer65-Ub releases the Ub-like (UBL) domain of Parkin from RING1 domain, partially activating Parkin ➃. Then, PINK1 phosphorylates the UBL domain at Ser65 ➄, which drives the phospho-UBL to rebind to the RING0 domain of Parkin to expose RING2′ catalytic site (Cys431) and fully activate Parkin ➅. On the other hand, phosphatase and tensin homolog long (PTEN-L) located at OMM dephosphorylates Ub to inhibit mitophagy, whereas PTEN in the cytosol suppresses mitophagy through targeting Rab7A, MFN2, or BNIP3.

Mitochondria are one of the essential organelles in eukaryotic cells, with critical functions including energy (ATP) production, cell survival/cell death, cell signaling, and immune response. Dysfunctional mitochondria are implicated in many pathological processes and diseases such as cell death, inflammation, neurodegenerative diseases, and cancer. Thus, removal of damaged mitochondria by mitophagy has been shown to be an important mitochondrial quality control mechanism to maintain the mitochondrial homeostasis. However, this process must be restricted to dysfunctional mitochondria. Excessive degradation of essential mitochondria will cause cell death (Ordureau and Harper, 2014; Shi et al., 2014; Guo et al., 2016; Sharma et al., 2019). In addition, during the mitochondria fission process, the membrane potential of healthy mitochondria is temporarily compromised (Twig et al., 2008), which possibly activates PINK1-Parkin pathway to remove healthy mitochondria. Therefore, the mitophagy machinery is orchestrated by key mitophagy effectors with reversible posttranslational modifications, such as phosphorylation and dephosphorylation, to determine a finely tuned mitophagic activity in response to diverse stresses (Figure 2).

We now appreciate that phosphorylation of Ub by PINK1 (pSer65-Ub) plays central roles in the regulation of Ub-dependent mitophagy pathway. pSer65-Ub levels are very low in healthy mitochondria, but dramatically increased after mitochondrial damage and also increased during aging or in PD patient brain, which highlights its roles in diseases (Fiesel et al., 2015; Hou et al., 2018). Although PINK1 is the only reported kinase to generate pSer65-Ub, pSer65-Ub could be detected in PINK knockout cells (Ordureau et al., 2014) and in PINK1-deficient yeast (Swaney et al., 2015), suggesting another kinase exists to phosphorylate Ub at Ser 65. However, the function of PINK1-independent pSer65-Ub remains largely unclear. Another question is whether pSer65-Ub can be involved in other selective autophagy, such as xenophagy, which shares several key factors with mitophagy, including TBK1, NDP52, OPTN, and SQSTM1.

Recent studies have indicated that PTEN family proteins are involved in the regulation of both PINK1-Parkin-mediated Ub-driven and BNIP3 receptor-mediated mitophagy. Some important questions need to be further addressed. First is how the cells determine the expression level of different PTEN isoforms to function under different conditions. Second is whether there is a specific recruitment of PTEN-L and PTEN to mitochondria in response to mitochondrial damage. Third and more importantly is whether PTEN isoforms can serve as molecular targets for development of novel interventional approaches in the regulation of mitophagy to benefit mitophagy-related human diseases.

## Author Contributions

LW and H-MS designed the outline of the review and wrote the draft of the manuscript. GL wrote part of the review.

## Conflict of Interest

The authors declare that the research was conducted in the absence of any commercial or financial relationships that could be construed as a potential conflict of interest.
